# Institutional transformations for authentic community engaged and participatory research

**DOI:** 10.1017/cts.2026.10715

**Published:** 2026-04-06

**Authors:** Nina Wallerstein, Milton (Mickey) Eder, Lori Carter-Edwards, Paige Castro-Reyes, Tanja Gangarova, Linda Sprague Martinez, Lloyd Michener

**Affiliations:** 1 Center for Participatory Research, College of Population Health, University of New Mexico Health Sciences Center, Albuquerque, NM, USA; 2 Department of Epidemiology, College of Public Health and Health Professions and College of Medicine, University of Florida Health Science Center, Gainesville, FL, USA; 3 Community Engagement and Government Affairs, Department of Health Systems Science, Kaiser Permanente Bernard J. Tyson School of Medicine, Pasadena, CA, USA; 4 Community-Campus Partnerships for Health, Raleigh, NC, USA; 5 National Monitoring of Discrimination and Racism (NaDiRa), DeZIM-Institute, Germany, Alice Salomon University of Applied Sciences Berlin, Germany; 6 University of Connecticut, Storrs, CT, USA; 7 Community and Family Medicine, Duke University CTSI, Durham, NC, USA

**Keywords:** Community/patient engaged research, community based participatory research, institutional transformations, academic health centers transformation, community voice/power

## Abstract

This special issue of the *Journal of Clinical Translational Science* on Institutional Transformation provides strategies to strengthen community and patient engagement in research in which collaborative knowledge creation is valued and centered in the history, knowledge, and evidence within communities. Recognizing the important role of academic health centers, schools of public health and research institutes in engaged research, the guest editors sought articles that challenged institutions to transform policies, practices, norms and structures towards power-sharing in research and towards commitment to sustainable research partnerships for health equity. While these articles were mostly written before the current context of large-scale terminations of grants and programs, this special issue recognized the well-founded historical distrust of communities in academic centers and the ongoing challenges of regaining trust in science. We first provide an historical context of institutional barriers and facilitators of engaged and participatory research and then review articles, including from the Engage for Equity PLUS national initiative. We end with recommendations for the field, as we recognize we still need to be self-critical about the structures that maintain academic dominance in research rather than valuing multiple ways of knowing and the importance of communities for authentic co-creation and leadership of research.

## Introduction

We face a new time. Since January 2025, the American funding landscape for research has changed dramatically with large-scale terminations of grants and fellowships across the U.S. Department of Human Health and Services. In addition to unfounded attacks on science, universities and community organizations have lost key funding, including research partnership awards, adding to community distrust of institutions. This reality is intensified in communities, especially those with long histories of disinvestment and discriminatory conditions, which have contributed to unequal health outcomes, and with community assets often remaining unrecognized.

Adding to the current crises, trust in institutions is low, with academic institutions especially not always being trustworthy partners. Despite the positive history of individual community participatory and patient engaged research projects, institutional barriers to community engagement are common, and include fragmentation of engagement efforts, bureaucratic financial systems, lack of coordination when connecting with communities, and insufficient support for interdisciplinary teams which include community partners. At a structural level, lack of commitment to inclusion of patient and community leaders in decision-making about research or about institutional priorities has been a particular challenge. The lack of recognition of the importance of community voice in decision-making is exacerbated by absent or inadequate connections of academic health centers to the many existing community networks and state infrastructures that seek to improve health for all.

Yet, despite these historic challenges and the current confusion, we have an opportunity to strengthen collaborations for greater connections to the public to regain trust in institutions and in science. Solution-oriented health disparities research remains an NIH priority (www.nih.gov/about-nih/nih-director/statements/advancing-nihs-mission-through-unified-strategy), while community engagement remains a central focus of the revised Clinical and Translational Science Awards (https://grants.nih.gov/grants/guide/pa-files/PAR-24-272.html). In this time of rapid change, academic institutions have an opportunity to build on community and state assets and to become committed partners and true anchor institutions in improving health for all populations [1]. Strategies are needed where expertise is not only among those with the highest academic degrees, but where expertise is a collective that centers and follows the voices of communities. This is a time in which academic institutions, more hamstrung in their own communications and funding, can take the lead from neighborhood and community organizations, patient advocacy groups, tribal programs and leaders, who bring their own knowledges, resiliencies, and histories of ceaseless organizing to improve the lives of those they serve.

This special issue of the *Journal of Clinical Translational Science* has sought to address questions of how to strengthen community and patient engaged research in which collaborative knowledge creation is valued and centered in the history, knowledge, and evidence within communities. Recognizing the important role of academic health centers, schools of public health and other research institutes in supporting engaged research, we sought articles that challenged these institutions to transform their policies, practices, norms and structures towards power-sharing in research and towards commitment to sustainable research partnerships for health equity. While these articles were mostly written before the current context, this special issue may be even more important if it can encourage all of us to recognize that community academic discordance must be acknowledged when present, and that innovations are needed to improve institutional policies and practices for authentic community participation, co-governance and leadership in research.

We first provide an historical context of community engaged and community based participatory research and of institutional conditions that have supported or been barriers to engagement. We then offer a review of the included articles with their strategies and directions for institutional transformation, including a set of articles from the Engage for Equity PLUS national initiative. We end with recommendations for future directions for the field, as we recognize that there is a long way to go in our abilities to be self-critical and to reflect on the structures that maintain academic dominance in research rather than valuing multiple ways of knowing and the importance of communities in co-creation of research. We now turn to exploring our histories further.

## History of engaged research and institutional context

Community engaged and community based participatory research approaches have gained tremendous momentum in the health sciences over the past three decades due to recognition of the historical rift between communities and academic institutions, named colloquially and professorially as the “town-gown divide.” The divide highlights an insider-outsider tension regarding the production of knowledge and culture, which fuels power imbalances and an academic elitism that suppresses non-academic ways of knowing [2]. Exclusionary policies and practices of university-based knowledge generation have played a role in transforming knowledge into a commodity [3]. The emergence of community-engaged research and community-based participatory research approaches has chipped away at the town-gown divide and some of the inequities it sustains. The inclusion of community engagement within the mission of translational science elevated the sentiment “nothing about us without us” along with recognizing the potential of those beyond the walls of academic institutions to participate in knowledge generation, implementation and diffusion [4,5].

In these decades, the ideal of engaging community voices in all aspects of the research enterprise has helped temper divisions between academic and community research roles by pursuing humanistic values within research [6]. Research partnerships have been shown to alter power relationships and challenge assumptions rooted in dominant sociocultural perspectives. It has served as a source of trust building, resource sharing, and civic participation [7]. Translational science has long contended that engagement in partnerships creates the potential to elevate community health concerns.

Support for community-engaged research and participatory research is evident in the historic commitments of the Centers for Disease Control and Prevention, the National Institutes of Health, foundations, and the Patient Centered Outcomes Research Institute. The commitment to academic-community partnership is further demonstrated within translational science by the increased attention to implementation and dissemination research [8,9]. The support for engagement has resulted in an expanding literature with evidence of partnering and participatory processes contributing to a range of outcomes, such as enhanced validity of research designs, increased recruitment, capacity-building and improved health [10–13].

National organizations have provided evidence of culturally-competent and socially-relevant engaged projects, such as from the National Academy of Medicine, (https://nam.edu/our-work/programs/leadership-consortium/assessing-meaningful-community-engagement/); the Association of State and Territorial Health Officials, (https://www.astho.org/topic/brief/best-practices-for-sustained-community-engagement-from-stretch/); the non-profit, Community Campus Partnerships for Health, (https://ccphealth.org/); as well as from community-academic collaborations that also include local and state health departments [14].

Institutional efforts have been furthered by the Association of American Medical College’s Center for Health Justice, which developed a toolkit on principles and strategies for institutions to demonstrate trustworthiness within their communities [15]. Trustworthiness as a form of critical self-awareness within institutional community engagement activities constitute an important addition to the third edition of the Principles of Community Engagement [16]. The National Academy of Medicine Task Force on Assessing Meaningful Community Engagement has promoted both trustworthiness and the pursuit of equity as indicators of meaningful community engagement [17]. These reports have gained traction, as the recently revised Notice of Funding Opportunity for the CTSA Awards calls for “…engaged and effective organizational governance and leadership structures that respond to needs of the community and input from advisory committees.”

On a local level, communities have been developing infrastructures to address their own priorities, often with foundation assistance, for example, (https://buildhealthchallenge.org/); while communities across the nation are mounting resistance to current attacks with mutual aid, organizing and advocacy to protect families. States also have been developing infrastructures to address systemic inequities, including community partnership development and data integration and analysis (https://www.astho.org/topic/public-health-data-research/environmental-scan/). Multistate health collaboratives have developed, such as the West Coast Health Alliance. Community Health Needs Assessments, required for charitable hospital organizations by the Affordable Care Act, as well as those by health departments provide opportunities for meaningful community engagement. These collaboratives present an opportunity for CTSAs to connect, partner, and scale at the local, state and regional levels.

Although progress has been made in enumerating engagement principles and processes, as mentioned above, there is much more to do, both in addressing historic institutional challenges as well as within the current national crises. There has been limited change in areas like institutional bill payment and partner compensation [18–22], yet institutional reward structures continue to tilt toward the researcher. Universities are expanding support for community partnerships, but their conceptualization of team science demonstrates only a marginal focus on incorporating community members and their perspectives [23]. Advisory boards have proven an acceptable initial strategy for integrating community into decision-making, but these boards are often project specific, provide feedback rather than decisions, and often are not sustained after the grant ends. Even successful partnerships have not led to the integration of community perspectives within institutional decision-making practices, let alone to new co-governance structures. As community and state efforts take priority for improving health, given the retrenchment underway in federal efforts, the institutional management of engagement becomes increasingly important to fulfilling the promise of translational science [24].

The commitment to bridging town-gown differences and reducing academic-community hierarchies through meaningful and enduring partnerships requires an embrace of disruption and uncertainty. Partners must acknowledge exclusion and mistrust where it persists. Assumptions embedded within societal norms and institutional practice that sustain inequity must be identified and confronted. Community benefit needs to be re-imagined as a primary goal. We see this collection appearing within a historical moment of institutional reflection on engagement, partnership, collaboration, resource-sharing, co-ownership and translation of data into results, sustainability and equity.

## Review of articles

The articles in this special collection expand the literature on best practices and outcomes from project-based community engagement to institutional practices and policies which can be starting points for needed change, such as funding the time of community partners so they can participate commensurate with academic partners [19,25–29], and which can occur through microgrants [25] or vouchers, [26,30]. These practices raise important questions about how participation and partnerships can be sustained beyond a specific grant or project [26,31]. Another starting point is identifying key people in the academic institution and key community organizations and people in engaging them in co-planning and supporting change, as having leadership support, internal change agents, diverse staff and community partners are best practices and frequently noted as part of change [19,26–29,31–33]. Academic investigators, especially ones new to community engaged research, can benefit from education about the history and norms of the local communities, and how trusted partnerships can be built [26,33]. Strengthened communications and transparency of information can also be a key element of change, as the complexities of academic health systems, and of communities, may not be well understood [26,28,29,31,33,34].

A deeper structural approach is identifying and changing academic systems and infrastructures so that they build and support effective and sustained community partnerships. Such larger efforts consider financial and administrative policies that prioritize community [34,35]; co-governance structures for community members on committees, rather than limiting community input to advisement [26,29,33,35]; taking the time to fund new partnerships, rather than requiring partnerships to be developed for pilot funds; [29] creating community-placed research spaces [30,35]; building in language justice and translation [26,27,34,35]; and respect for different cultural norms and knowledge [26,35] as essential to sustained, trusted engagement. These larger efforts require leadership support, as they challenge decades of accepted practice, and raise considerations that have historically not been acknowledged or seen as germane [26,28,29,32,36].

Seven articles in this special issue come from Engage for Equity PLUS (E2PLUS), a national PCORI engagement award (#21068, 2021-2023), led by the University of New Mexico Center for Participatory Research (UNM CPR). Developed to confront these institutional challenges, UNM CPR partnered with three very different Academic Health Centers (AHCs)–Stanford School of Medicine, Morehouse School of Medicine, and the Fred Hutch/University of Washington Cancer Consortium. The goal was to better understand AHC infrastructure contexts of barriers and facilitators and to assess the feasibility of the E2PLUS intervention to promote institutional transformations towards collaborative co-governance and enhanced community/patient engagement in research.

E2PLUS strategies included use of evidence-based E2 tools developed from cycles of NIH funding since 2006 to identify and strengthen best partnering practices at the project level associated with outcomes [37,38] (https://engageforequity.org). E2PLUS sought to scale up impact to the institutional level through engagement of multi-directional coaching of champion teams (with half academics and half community/patient advocate members), workshops for a broader AHC audience, and survey and qualitative data as mechanisms for advocacy to top leaders, supported by a community of practice across institutions. E2 tools in workshops included the River of Life to co-construct histories and contexts of engagement, the CBPR Model to envision practice and policy targets of change, and ongoing cycles of Freirian praxis of collective dialogue and action in workshops, champion team meetings, and advocacy strategies [39].

Four E2PLUS articles provide analyses across institutions, with each champion team producing their own article with lessons learned. Two articles showcase aggregated data of existing policies and practices, with one providing academic and community responses from a new Institutional Multi-Sector Survey (IMSS) [40]; and the second, using mixed-methods to identify key barriers across the AHCs, such as policies which maintain power imbalances, as well as key facilitators of accountability and communication [33]. Muhammad et al (2025) present patient/community perspectives on realigning institutions with community priorities [34]. Sanchez-Youngman et al (2025) highlight how the E2PLUS intervention provided strategies and data for champion teams to catalyze advocacy for changes to top leaders [36]. Each champion team shared their own change strategies, such as addressing IRB and post-award barriers at Stanford [41]; promoting a new Office of Patient Engagement within the Fred Hutch/UW consortium [42]; and leveraging E2PLUS for system improvements in equity, building on what existed within Morehouse [43].

A current PCORI Science of Engagement award (SOE 33314, 2024–2027) at the UNM Center of Participatory Research seeks to challenge the field further by testing E2PLUS within eight new Academic Health Centers and adding a new patient/community action group (PCAG) strategy to expand the goal of trustworthiness to the outcome of enhanced ***leadership*** and co-governance of patient/community partners in research through actions to transform the inter-connected institutional and community engagement eco-system.

In addition, a recent *American Journal for Public Health* issue on Meaningful Community Engagement to Reduce Burden of Chronic Disease includes descriptions of innovations that have occurred as a result of academic-community partnerships, including enhanced tools to assess engagement, expanded tools for sharing lived experiences [44], revised use of data that is shared across the partnerships [45], community assessments of barriers [46], cluster mapping of strategies to promote translation of research findings into community [47], and system change [48], including redesigned meetings that include community partners and highlight community perspectives, and training in research for community partners [49].

## Discussion and conclusion

This special issue has brought together in one collection examples of institutional changes that highlight the importance of transformation of research infrastructures, especially in this time of disruption and change. In today’s world, the potential for a resurgence of infectious disease is real, despite the recent global experience with COVID-19. Not only did the basic science of mRNA vaccines save millions of lives by preventing severe disease and death, but the complementary *science of community engagement* was critical for increasing vaccine uptake and now continues with research across the nation that supports communities in their own health priorities and healing. With science being attacked and defunded and policies being enacted that will reduce access to health and health care, which are already poorly distributed in the U.S., we need actions across the fields of public health, medicine, nursing, social science and clinical translational science that will rebuild trust in science and academia to be transparent and responsive to community concerns and inclusive of community strengths.

We hope these articles can become a catalyst for greater attention to eco-system-wide structural changes that support the science of engagement and the practice of equitable partnerships and community and patient leadership in research. We acknowledge the remarkable work that has been done within engaged research over the last decades, yet we also acknowledge the gaping needs. With many calling attention to the gap in trust between academic institutions and communities, we need to go to the next level and address the structural causes of this ongoing lack of trust, and the need for institutions to be trustworthy.

One key translational gap is the need to go beyond creating community advisory boards, but instead to go to the community and listen, to become connected to the fabric of communities we serve, and to become deserving of their trust through practices that support community co-ownership of research processes and outcomes. This requires two inter-related strategies: (1) dismantling institutional structures that duplicate, conflict or repress community structures and knowledge; and (2) shifting from engagement with the community to deference to and respect for the community in working with their organizations and priorities.

What does dismantling institutional structures mean? It can mean a sustained commitment to self-reflection—particularly regarding the dominance of Western academic institutions in shaping what is considered legitimate knowledge. This dominance often mirrors the overrepresentation of white scholars, many of whom may not share the lived experiences of the communities they serve. As José Medina (2018) argues, we all can engage with our shadow selves, including a critical confrontation with our knowledge gaps and our complicity in structures that uphold (white) ignorance, in order to foster self-awareness and epistemic responsibility and justice [50]. We also can learn how to pair collective reflection with action in praxis reflection/action cycles for transformation, articulated by Paulo Freire, to gain trustworthiness [37,51,52]. In research practices, it can mean recognition that community involvement can be tokenistic if there is no decision-making power, particularly at an institutional level. As Tuck and Yang (2014) state, there is often the misconception that by simply bringing community members to the table, “the ethical issues of representation, voice, consumption, and voyeurism are resolved.”[53, pg. 230]. While we want to support patient and community advisory committees as a critically important engagement practice, dismantling structures can mean institutions going beyond committees to adopt co-governance agreements for community research projects, where data justice agreements are normalized, with data owned by communities. It can also mean redirecting institutional funds (such as from endowments, and/or direct and indirect funds) as bridge funding or beyond episodic grants, to support enduring partnerships so the work can continue, which may support community-based organizations to not lose key staff essential to sustaining equity efforts, especially needed in this time.

What does shifting towards deference to community and patient organizations mean? It can mean supporting community research hubs outside the academy where community based organizations set research priorities and provide ethics oversight or guidance to research in their communities. Community ethics boards are growing, following the experience of Tribal Institutional Review Boards, in which community benefit is an essential part of ethics review. It can mean learning to reciprocally braid western and community/culture-centered knowledge to be mutually respectful. It can mean paying attention to best partnering practices, such as equitable sharing of resources to ensure community benefit [54,55]. Prioritizing communities does not imply that we advocate returning to the victim-blaming of the 1970s and 80s, during which communities were blamed for their problems without recognizing the role of disinvestment in public resources. Instead, the strategy of funding community, tribal and patient organizations directly can be a way to shift power towards communities in research. PCORI and foundation funding for patient and community organizations; long-term NIH Native American Research Centers for Health (NARCH) funding to tribes; and restored partial NIH funding for community-based organizations to lead structural change research, (previously known as ComPASS and now an initiative of Community-led Research Projects), have begun to give credence to the importance of community power and ownership of research [56,57].

The examples in this special issue demonstrate some of our capacities to engage in system changes. Our field already offers a strong literature of complementary engagement models, nationally-recognized principles of engagement, published best partnering practices leading to outcomes, and a growing community engaged translational science. We have models of capacity building and consultation services for research projects, so it may be time for a consultative service for institutions and their community engagement cores, whether in CTSAs, comprehensive cancer centers, or university engaged scholarship centers.

But regardless of where change begins, or when a more systemic change process is used, the path forward needs to be flexible and responsive to the shifting environment within academic health systems and the wide range of communities with which they engage. The current context needs to be acknowledged as an enormous challenge, with the need to recognize the pain, fear and struggle for survival among communities across the country. With diverse communities facing threats of deportation, individuals are not only afraid to send children to school or seek medical care, this reality may also hamper willingness to participate in research. This time is a catalyst, therefore, for researchers to break down silos within their institutions to create coalitions with community and patient partners working together for change, supported by academic and medical leaders who increasingly recognize engagement strategies are needed to rebuild trust in health sciences (https://nam.edu/event/building-trust-in-health-science-roundtable-learning-leading-together/) [52,58]. This is a time for learning how communities are resisting, and for academics to learn from community organizers, as identified by the Health and Power Organizing Project, (https://uwphi.pophealth.wisc.edu/public-health-tools-and-resources/health-and-power-organizing-project-hpop/) [59]. It’s a time for learning how to reach out to trusted state or local leaders and to ask how academic institutions can support their work. With many nations still looking to the U.S. for our innovations in CBPR and community and patient engaged research, we have an opportunity to find and show a path forward.

As the many articles in this special issue demonstrate, there is no one right answer or approach. What matters is beginning and then continuing the journey, “walking the talk,” and challenging academic structures and processes to support partnerships that earn trust through co-governance, equitable resource- and power-sharing, and leadership from and with communities.
